# Alpha-sitosterol: a new antiviral agent produced by *Streptomyces misakiensis* and its potential activity against Newcastle disease virus

**DOI:** 10.1186/s12917-023-03875-y

**Published:** 2024-02-27

**Authors:** Rewan Abdelaziz, Yasmine H. Tartor, Ahmed B. Barakat, Gamal EL-Didamony, Marwa M. Gado, Mohamed Samir A. Zaki, Refaat A. Eid, Hanaa A. El-Samadony

**Affiliations:** 1 Department of Microbiology, Faculty of Science, Ain Shams University, Cairo, 11566 Egypt; 2 Department of Microbiology, Faculty of Veterinary Medicine, Zagazig University, Zagazig, 44511 Egypt; 3 Department of Botany and Microbiology, Faculty of Science, Zagazig University, Zagazig, 44519 Egypt; 4 Anatomy Department, College of Medicine, King Khalid University, P.O. Box 62529, Abha, Saudi Arabia; 5 Department of Pathology, College of Medicine, King Khalid University, Abha, 12573 Saudi Arabia; 6 Department of Poultry, Animal Health Research Institute, Dokki, Agriculture Research Center, Giza, 44511 Egypt

**Keywords:** Newcastle disease virus, *Streptomyces misakiensis*, α-Sitosterol, Bioactive antiviral agent, Hemagglutination, Histopathology

## Abstract

**Background:**

Newcastle Disease Virus (NDV) causes severe economic losses in the poultry industry worldwide. Hence, this study aimed to discover a novel bioactive antiviral agent for controlling NDV. *Streptomyces misakiensis* was isolated from Egyptian soil and its secondary metabolites were identified using infrared spectroscopy (IR), gas chromatography–mass spectrometry (GC–MS), and nuclear magnetic resonance (NMR) spectroscopy. The inhibitory activity of bioactive metabolite against NDV were examined. Three experimental groups of 10-day-old specific pathogen-free embryonated chicken eggs (SPF-ECEs), including the bioactive metabolite control group, NDV control positive group, and α-sitosterol and NDV mixture-treated group were inoculated.

**Results:**

α-sitosterol (Ethyl-6-methylheptan-2-yl]-10,13-dimethyl-dodecahydro-1H-cyclopenta[a]phenanthren-3-ol), a secondary metabolite of *S. misakiensis*, completely inhibited hemagglutination (HA) activity of the NDV strain. The HA activity of the NDV strain was 8 log^2^ and 9 log^2^ for 0.5 and 0.75% RBCs, respectively. The NDV HA activity for the two concentrations of RBCs was significantly (*P* < 0.0001) inhibited after α-sitosterol treatment. There was a significant (*P* < 0.0001) decrease in the log 2 of HA activity, with values of − 0.500 (75%, chicken RBCs) before inoculation in SPF-ECEs and − 1.161 (50%, RBCs) and − 1.403 (75%, RBCs) following SPF-ECE inoculation. Compared to ECEs inoculated with NDV alone, the α-sitosterol-treated group showed improvement in histological lesion ratings for chorioallantoic membranes (CAM) and hepatic tissues. The CAM of the α-sitosterol- inoculated SPF-ECEs was preserved. The epithelial and stromal layers were noticeably thicker with extensive hemorrhages, clogged vasculatures, and certain inflammatory cells in the stroma layer in the NDV group. However, mild edema and inflammatory cell infiltration were observed in the CAM of the treated group. ECEs inoculated with α-sitosterol alone showed normal histology of the hepatic acini, central veins, and portal triads. Severe degenerative alterations, including steatosis, clogged sinusoids, and central veins, were observed in ECEs inoculated with NDV. Mild hepatic degenerative alterations, with perivascular round cell infiltration, were observed in the treated group.

**Conclusion:**

To the best of our knowledge, this is the first study to highlight that the potentially bioactive secondary metabolite, α-sitosterol, belonging to the terpene family, has the potential to be a biological weapon against virulent NDV. It could be used for the development of innovative antiviral drugs to control NDV after further clinical investigation.

**Supplementary Information:**

The online version contains supplementary material available at 10.1186/s12917-023-03875-y.

## Background

Newcastle disease (ND) is a highly consequential viral disease affecting the respiratory, nervous, and digestive systems of poultry, caused by the Newcastle disease virus (NDV). It has a significant impact on the economy, resulting in financial losses owing to the high mortality rate of affected animals and subsequent condemnation of their carcasses [1]. Chickens are the domestic poultry species most susceptible to NDV, whereas waterfowl displays the lowest susceptibility [2]. NDV can cause a variety of damages to the organs of birds; in certain circumstances, the mortality rate can reach 90%. ND can have a wide range of symptoms including respiratory illnesses, diarrhea, neurological symptoms (such as anxiety, depression, trembling muscles, drooping wings, head and neck twisting, circling, and total paralysis), and egg drops, which are often associated with peritonitis in laying birds [3]. Many countries in Asia, Africa, and particular regions of North and South America experience frequent endemic outbreaks of highly pathogenic NDV strains in poultry [4]. ND referred to as Ranikhet infection and avian paramyxovirus serotype 1 (PMV-1), is of the utmost importance among the 11 recognized PMV serotypes in terms of its impact as a pathogen in chickens [3, 4]. NDV strains are classified into five pathotypes depending on the severity of the infection: velogenic viscerotropic, velogenic neurotropic, mesogenic, lentogenic, and asymptomatic enteric [2]. Although all NDV strains belong to the same serotype, antigenic and genetic diversity have been reported [2]. The most common NDV genotypes currently circulating globally are V and VII [4].

The classification of NDV isolates according to their degree of virulence, specifically as highly virulent (velogenic), moderately virulent (mesogenic), or low virulence (lentogenic), has been made more efficient for regulatory purposes. Infections caused by the lentogenic strains of NDV are commonly used as live vaccines. The clinical manifestations of ND vary from asymptomatic infections to severe morbidity and mortality [5]. The factors that influence ND outcomes include host susceptibility, viral pathogenicity, host age, host immunological state, and infection severity [5]. NDV is an obligatory intracellular pathogen that lives only in living cells. Therefore, they can be grown in embryonated chicken eggs (ECEs), which are cheap media, easy to perform, and accurate for demonstrating patterns of virus change in the host. NDV causes high mortality in embryos on the 2nd day post-inoculation, and hemorrhages on all parts of the embryo bodies [6]. Chickens can be readily infected through the inhalation of aerosols, as well as through the consumption of contaminated water or food. NDV transmission can occur through infected avian species, including chickens, as well as various other domestic and wild animals [7]. The primary modes of virus transmission between poultry flocks encompass the migration of infected birds and the dissemination of viruses, particularly through infectious fecal matter, transportation of individuals, contaminated equipment, and waste materials [8].

Natural products play a crucial role in the discovery and development of novel pharmaceuticals, serving as valuable sources of new medications and prototypes for the synthesis of innovative drugs [9, 10]. Microorganisms are responsible for the synthesis of numerous natural product drugs [9]. Actinobacteria are significant bacterial taxa known for their ability to synthesize valuable secondary metabolites [11]. Actinobacteria, both historically and currently, serve as significant reservoirs for the discovery and development of novel pharmaceutical agents. The attention given to *Streptomyces* spp. has been significant because of their extensive and potent biological activities, specifically antiviral, antifungal, and anticancer properties [3, 9]. These metabolites are comprised of oxygenated monoterpenes, diterpenes, and fatty acids. Oxygenated terpenes exhibit antiviral efficacy against a range of viruses, encompassing coronaviruses and human immunodeficiency virus [9]. *S. misakiensis* is a bacterial species belonging to the genus Kitasatospora that has been isolated from soil [9]. *S. misakiensis* can generate bioactive secondary metabolites, including tobramycin A, tobermycin B, misakimycin, and endothelin receptor antagonist BE-18257B [11]. Recently, the phytoestrogen β-sitosterol and its O-glycoside derivative exhibit potent in vitro antiviral activity against influenza A viruses by blocking the hemagglutinin surface protein’s active binding sites and demonstrating inhibitory effects against replication through interacting with influenza [12].

Hence, this study was designed to ascertain the in vitro antiviral potential of secondary metabolites of *S. misakiensis* against NDV. Furthermore, the scope of this study included the characterization of *S. misakiensis* secondary metabolites using gas chromatography-mass spectrometry (GC-MS), infrared spectroscopy (IR), and nuclear magnetic resonance spectroscopy (NMR).

## Results

### Identification of *S. misakiensis* bioactive metabolites

The isopropanol extract derived from *S. misakiensis* metabolites was subjected to thin-layer chromatography (TLC) analysis. Ten fractions of the metabolite extracts were analyzed. The antiviral activities of the fractions were assessed using a hemagglutination test to validate the efficacy of the active chemical compounds. Additional identification techniques have been employed for the effective fraction R5, including GC-MS, IR spectroscopy, and NMR spectroscopy.

**Fig. 1:**
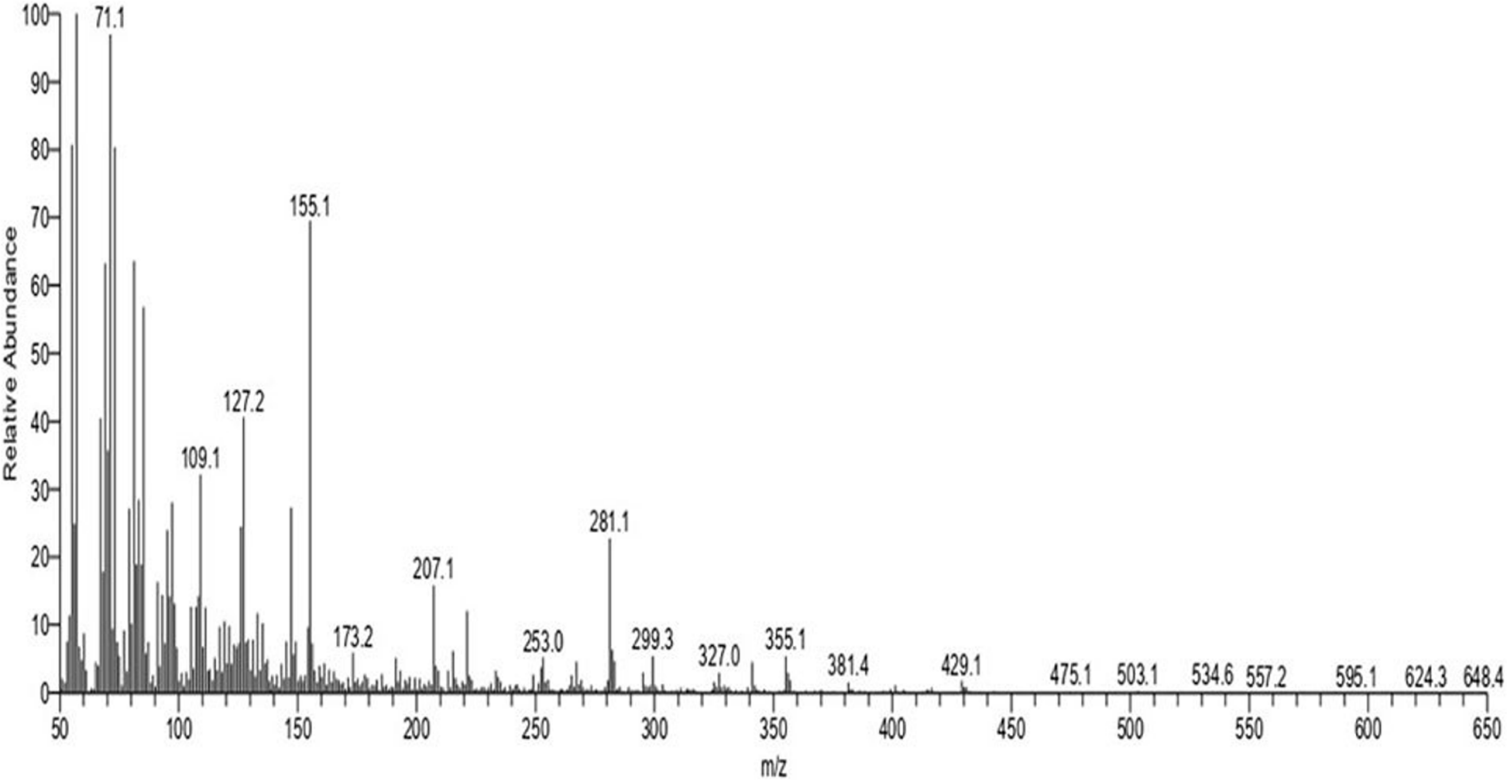
GC-MS analysis of α-sitosterol using isoprobanol as the solvent

As depicted in Fig. 1, the GC-MS analysis of *S. misakiensis* metabolites revealed the predominant presence of α-sitosterol (Ethyl-6-methylheptan-2-yl]-10,13-dimethyl-dodecahydro-1H-cyclopenta[a]phenanthren-3-ol).

**Fig. 2:**
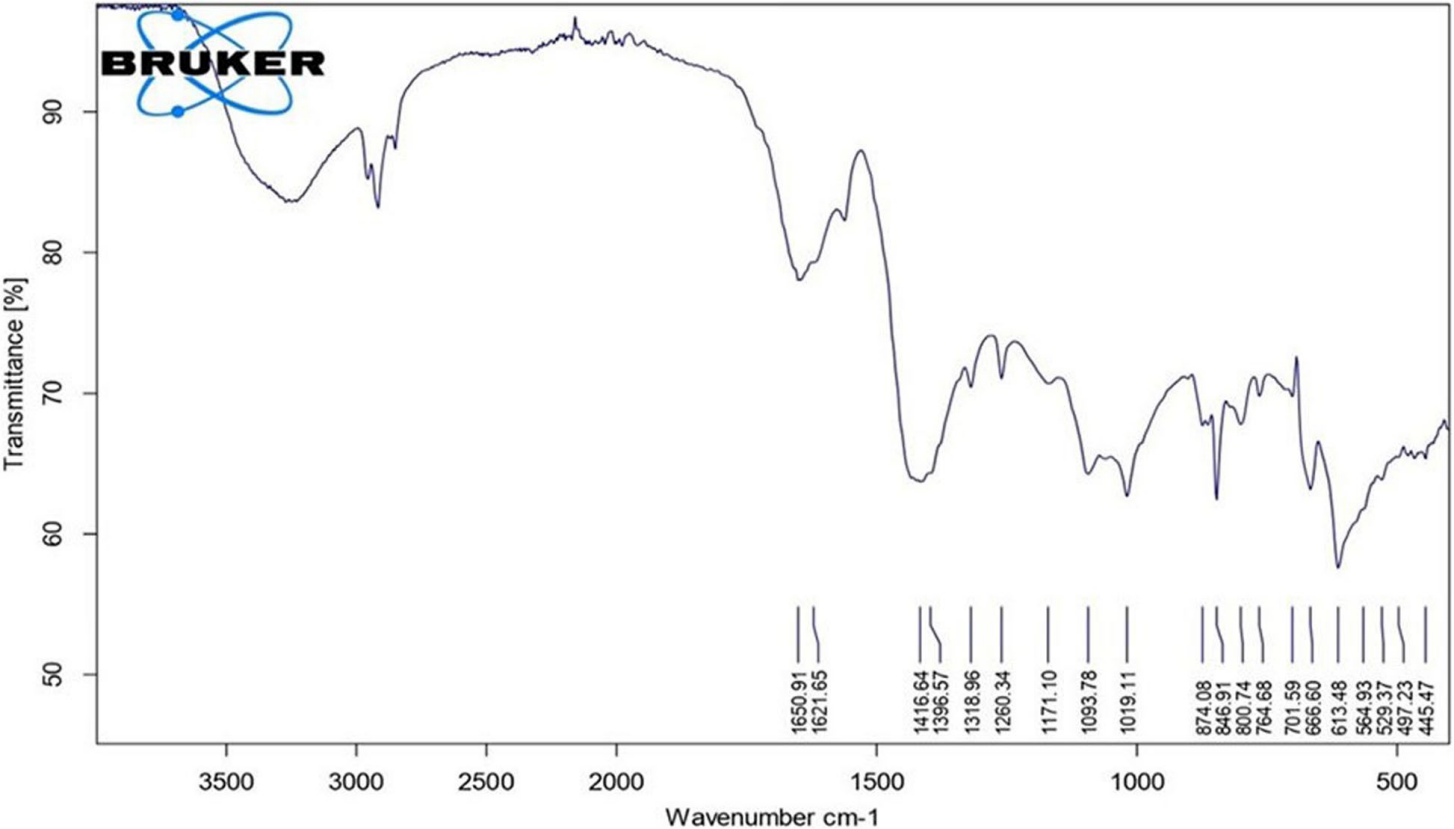
IR spectrum of α-sitosterol

The IR spectrum of α-sitosterol exhibited distinct peaks at 1650, 1621, 1416, 1171, 1019, and 445 cm-1, corresponding to the functional groups of phenol, hydroxyl (Ʋ OH), aliphatic carbon-hydrogen bonds (Ʋ C-H), carbonyl (Ʋ C═ O), ester, and benzene derivative with a carbon-carbon double bond (Ʋ C═ C), respectively, as illustrated in Fig. 2.

**Fig. 3:**
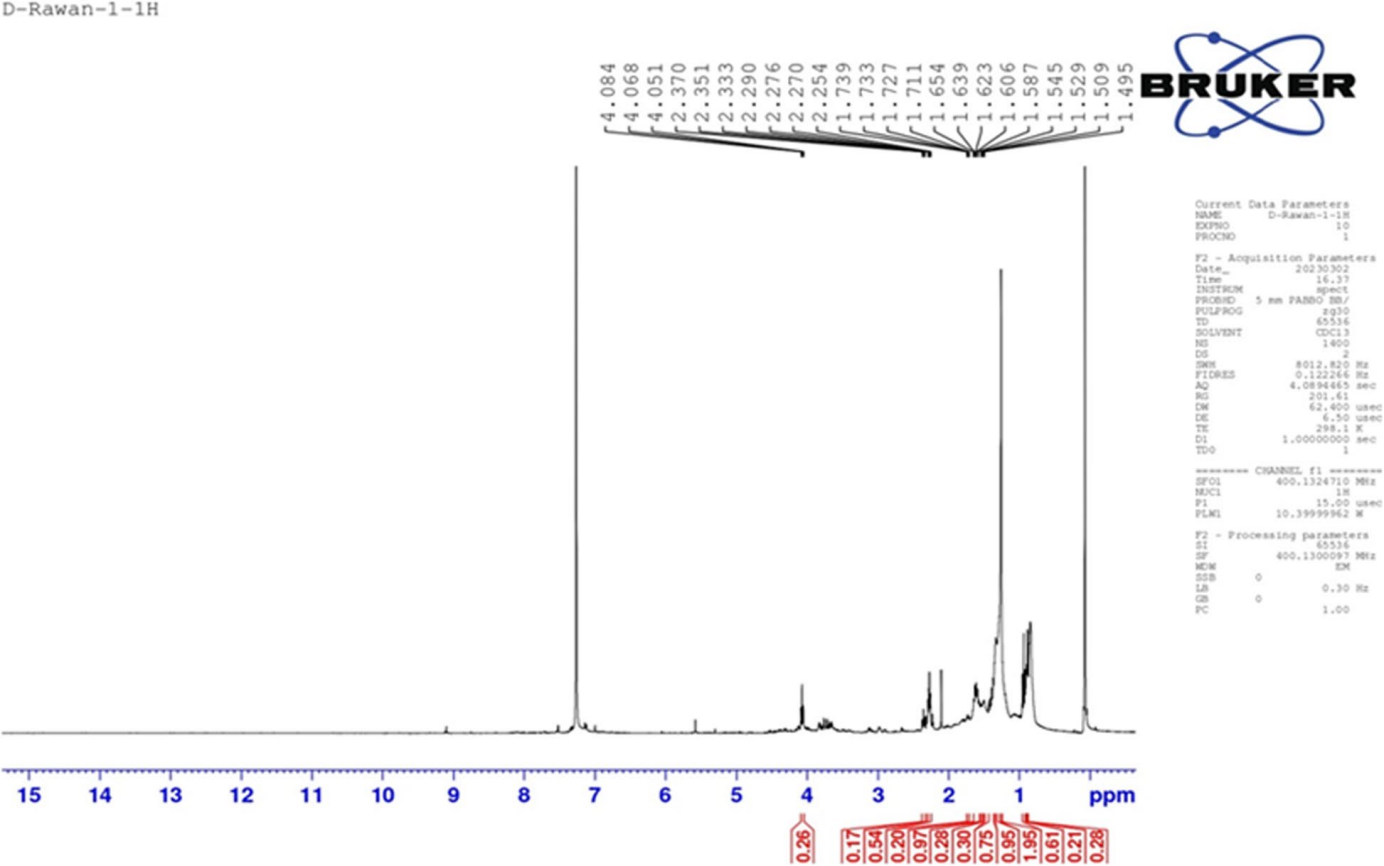
NMR spectrum of the derivatives of α-sitosterol

Figure 3 shows the NMR spectra at 8.09, 4.26, 3.17, 2.30, and 1.62 ppm. Allylic, alcohol, OH, and aldehyde groups, demonstrating that this antiviral was α-sitosterol.

### Hemagglutination activity of NDV with α-sitosterol

Erythrocytes cells were used to determine the impact of this bioactive agent on the hemagglutination (HA) activity of the virus and its adhesion to chicken red blood cells (RBCs). As revealed in Additional Fig. S1, α**-**sitosterol treatment caused chicken erythrocytes to precipitate, indicating that virus agglutination was prevented. This indicates that α-sitosterol prevents viral attachment to chicken RBCs (in vitro) and subsequently spreads to the infected host cells. The NDV strain had an HA activity that varied between 7 and 9 log2 with concomitant RBCs concentrations of 0.5 and 0.75%. The NDV strain and α-sitosterol mixture completely inhibited NDV HA (zero) in vitro, but after SPF-ECE inoculation, the agglutination activity returned to a low degree (Table 1).

**Table 1:** Hemagglutination activity of NDV after mixing with α-sitosterol

α-Sitosterol	RBCs concentration (%)	Hemagglutination activity of NDV (log_2_)				β	*P*-value	Means (log_2_) ± SD
		V/V	1/2	1/4	1/8			
**Before SPF-ECE inoculation**	0.5%	Zero	Zero	Zero	Zero	–	–	–
	0.75%	2^2^	Zero	Zero	Zero	- 0.500	0.001	1.00 ± 0.00
**After SPF-ECE inoculation**	0.5%	2^7^	2^5^	2^3^	Zero	−1.161	0.001	1.953 ± 0.521
	0.75%	2^9^	2^7^	2^5^	Zero	−1.403	0.001	2.564 ± 0.343

*PBS* Phosphate buffered saline: *SPF-ECE* Specific pathogen free-embryonated chicken egg: β: the regression coefficient. Zero indicates precipitation of RBCs. *SD* standard deviation: HA of original strain 8 log^2^ and 9 log^2^ with RBCs 0.5 and 0.75%, respectively. The *P-*value of NDV after being treated α-Sitosterol was revealed significant effects (*P* < 0.0001) on the hemagglutination activity with both two concentrations of RBCs (0.5 and 0.75%)

The HA activity of the NDV strain was 8 log^2^ and 9 log^2^ for 0.5 and 0.75% RBCs, respectively. The NDV HA activity for the two concentrations of RBCs (0.5 and 0.75%) was significantly (*P* < 0.0001) inhibited after treatment with α-sitosterol.

### Hemagglutination activity of NDV following injection of SPF-ECE and the impact of α-sitosterol

The HA activity of the NDV in allantoic fluid collected following combination inoculation in SPF-ECE is presented in Table 1 and Additional Fig. S1. There was a significant (*P* < 0.0001) decrease in the log 2 of HA activity, with values of − 0.500 (75%, RBCs) prior to specific pathogen-free embryonated chicken eggs (SPF-ECE) inoculation and − 1.161 (50%, RBCs) and − 1.403 (75%, RBCs) following SPF-ECE inoculation. HA activity of NDV was decreased after mixing with α-sitosterol. This indicated that α-sitosterol inhibited the binding of NDV to RBCs.

### Histopathological changes in ECE liver and chorioallantoic-membranes

**Fig. 4:**
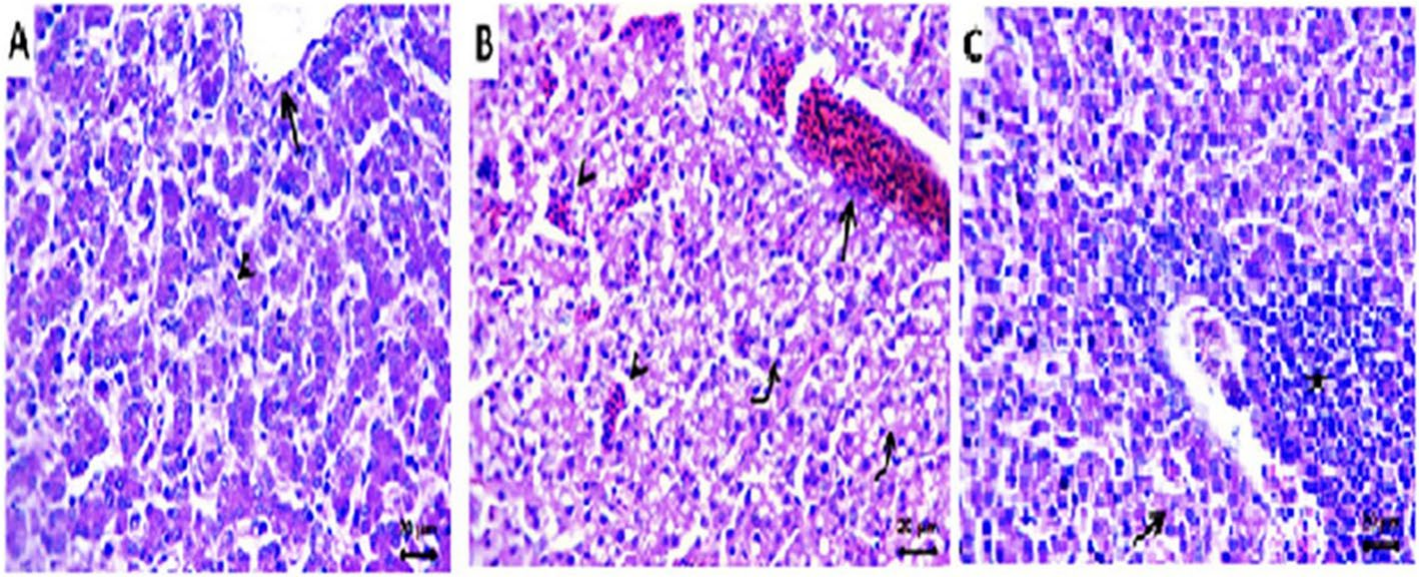
Representative photomicrograph of H&E-stained sections of ECE liver. **a**: Normal histology of the liver parenchyma (arrowhead), sinusoids, and central vein (arrow) in the control group. **b**: Extensive steatosis (curved arrows), congested sinusoids (arrowhead), and central vein (arrow) in the control positive group. **c**: The treated group has a few degenerate hydropic hepatocytes (arrow) and perivascular round cell infiltrations (star). Scale bar 20 μm

The effects of α-sitosterol and histological alterations in the ECE liver were investigated following egg inoculation. The group that received only α-sitosterol showed normal liver parenchyma, sinusoids, central veins, and portal triads (Fig. 4 A). The livers of the control group (inoculated with NDV) displayed severe degenerative alterations, primarily steatosis, as shown by distinct cytoplasmic vacuoles with centrally or peripherally positioned nuclei. Central veins and clogged sinusoids were also visible (Fig. 4 B). Mild hepatic degenerative alterations with perivascular round cell infiltrations were visible in the livers of the treated group (Fig. 4 C).

**Fig. 5:**
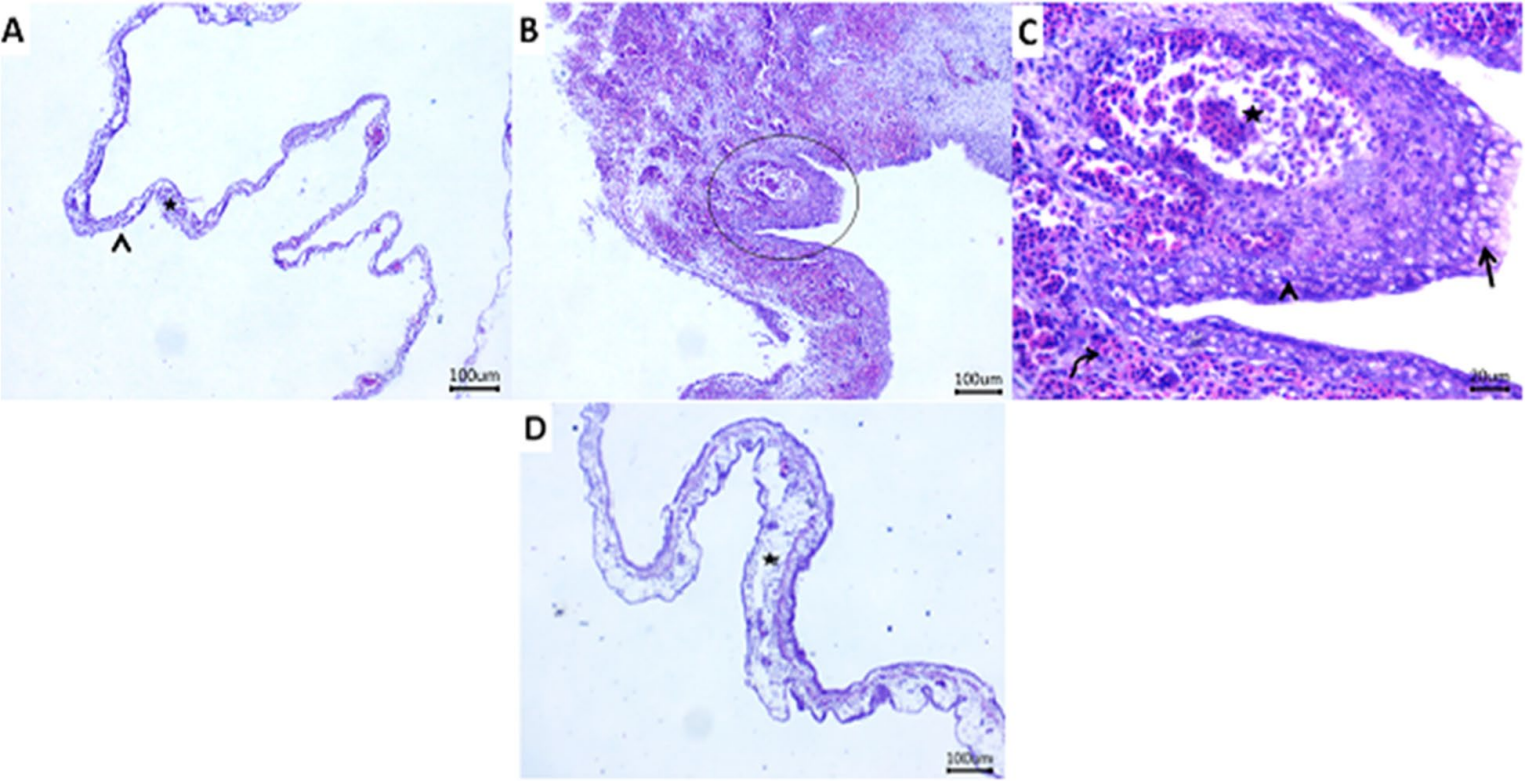
Representative photomicrograph of H&E-stained sections of ECE CAM. **a**: Normal chorioallantoic epithelium (arrowhead) and connective tissue layer (star) in α-sitosterol group. **b**, **c**: Extensive hemorrhages (curved arrow), congested vasculatures (star), and some inflammatory cells within the stroma layer. Additionally, hyperplastic vacuolated epithelium (arrow) with intracytoplasmic inclusions (arrowhead) within the epithelial layer in the control positive group (scale bar 100, 20 μm). C: Edema and inflammatory cells infiltrate (star) with a moderately thickened chorioallantoic membrane in the treated group (scale bar 100 μm)

The chorioallantoic epithelium and connective tissue layer were normal in the group that received α-sitosterol (Fig. 5 A). Epithelial and stromal layers were noticeably thicker in the NDV group. Extensive hemorrhage, clogged vasculatures, and certain inflammatory cells were observed in the stromal layer. A hyperplastic vacuolated epithelium with intracytoplasmic eosinophilic inclusions was also visible in the epithelial layer (Figs. 5 B and Figs. 5 C). Owing to edema and inflammatory cell infiltration, the chorioallantoic membranes (CAM) in the treated group were moderately thickened (Fig. 5 D). A semi-quantitative lesion score of liver and CAM alterations is summarized in Table 2**,** in which marked histopathological alterations in the hepatic and CAM tissues were observed in the group inoculated with NDV alone in comparison with the group inoculated only with α-sitosterol. The treated group (NDV with α-sitosterol) showed amelioration of histological lesion scores in comparison with the NDV-inoculated group.

**Table 2:** Scores of lesions in liver and chorioallantoic membrane tissues of specific pathogen free-embryonated chicken eggs among different experimental groups

Tissue	Lesions	*α-sitosterol control group	NDV control positive group	α-sitosterol and NDV mixture treated group
**Liver**	Hydropic degenerated cells	0	2	1
	Steatosis	0	3	1
	Round cells infiltrations	0	2	1
	Congested vasculatures	0	3	0
**Chorioallantoic membrane**	Hyperplastic epithelium	0	3	1
	Vacuolated epithelium	0	2	1
	Hemorrhages	0	2	0
	Congested capillaries	0	3	1
	Edema	0	2	2
	Inflammatory cells infiltrate	0	2	2

*Examined specific pathogen free-embryonated chicken egg (SPF-ECE) = 15 ECEs /group. Number of examined fields (five fields/ ECE, X100). The lesions were graded by estimating the percentage area affected in the entire section: Lesions score system was as follows: 0 = absence of lesion, 1 = 5–25%, 2 = 26–50%, and 3 = >50%

## Discussion

ND is one of the most significant avian diseases and causes severe financial loss [13]. In an outbreak, infected and susceptible birds are culled to contain transmission of the disease [13]. Therefore, searching for a product with antiviral activity is essential to control such a problem. It is crucial to conduct research that focuses on the development of new compounds to advance drug-based therapies against viral infections [14, 15]. This study is the first to report the antiviral activity of α-sitosterol against NDV. Actinobacteria produce secondary metabolites with antibacterial, antifungal, antiparasitic, antiviral, and anticancer activities that are structurally effective and functionally diverse [16, 17]. A serious issue is that commercially marketed vaccinations do not always have the desired effects [18]. A potential solution to this expanding issue is the development of innovative antiviral drugs that employ ingredients from currently available natural products [5, 13, 19, 20].

Structural analyses of the *S. misakiensis* bioactive metabolite showed a unique molecular structure (Figs. 1–3). This structure matched that of α-sitosterol, according to the results of NMR, IR, and GC-mass analyses. These results mirror those of Joo [21], who verified the structure of α-sitosterol. This is supported by the fact that sitosterol possesses antiviral action against NDV owing to its functional groups. Recently, Xie et al. [22] reported that α-sitosterol could replace oxytetracycline as a growth-promoting additive in broiler chicken feed and has proven to be effective against NDV.

Sitosterol has been linked to a variety of biological effects in the context of pharmaceutical research, including anticancer, anti-inflammatory, androgenic, antioxidative, antidiabetic, angiogenic, anorexic, antileukemic, chemoprotectant, and neuroprotective [20, 21, 23]. The steroid compound β-sitosterol shows some of the most effective activities against influenza A viruses by suppressing spike glycoproteins [23, 24]. Steroids may have a marked and considerable effect on the virus directly in a cell-free state by (i) influencing many stages of the viral replication cycle, including viral adsorption and replication and (ii) inhibiting replication by binding and blocking the active binding sites of hemagglutinin surface proteins. These ways for this chemical as antiviral occupy the viral adsorption onto the host cell receptor thus prevent virus adhesion, later inhabit the viral replication inside the host cells, and these targeting the viral particles away from the cell (cell free viricidal effect) [20, 23].

β-sitosterol can also be used to improve immunity against the SARS-CoV-2 infection and to limit the viral invasion into the host cell. The consumption of β-sitosterol and other phytosterols can modify immunity, which is necessary to combat COVID-19 [23, 25].

Bouic and Lamprecht [26] demonstrated the antioxidant and immune-stimulating activities of β-sitosterol, a phytosterol compound that appears to focus on Th1 and Th2 T-helper lymphocytes, normalizing their function and enhancing T-lymphocyte and natural killer cell activity. In many disease processes involving persistent immunological-mediated disorders, such as chronic viral infections, TB, rheumatoid arthritis, allergies, cancer, and autoimmune illnesses, the restoration of these immune parameters may be beneficial [27]. Many studies have examined the viricidal activity of β-sitosterol compounds by examining their effect on the hemagglutinin protein [28]. A simulated investigation indicated the capacity of this compound to target the surface of the spike glycoprotein on the viral particle against SARS-CoV-2, proving that sitosterol possesses inhibitory effects against the tested virus [29].

Beta-hydroxy steroid and delta (7)-sterol, α-sitosterol are chemical structure component of cyclic (Z)-24-ethylidenelophenol [19]. Owing to its structure, which includes conjugated double bonds, hydroxyl, carbonyl, and an amide group, α-sitosterol could potently inhibit viral disease. This is done by partially inhibiting the binding of virions to cells, which is dependent on glycoprotein C, and by inhibiting the processes that take place after the virus has been bound to cells [30].

A sialylated molecule that can prevent viral attachment to cellular receptors may limit the early stages of viral infection, in contrast to neuraminidase (NA) inhibition, which is believed to primarily act by limiting the release of new virions from virus-infected cells. Additionally, Neuraminidase inhibitors **(**NAI) must be administered early in the infection because as it develops, it becomes less effective [31].

α-sitosterol has a significant potential antiviral impact on the NDV strain. The results demonstrated full suppression of NDV HA in vitro*;* however, following SPF ECE inoculation, a reduction in log2 HA was predicted to be − 0.60 for each unit increase in α-sitosterol mixed with NDV (Table 1). This indicates the capacity of α-sitosterol’s to prevent the binding of NDV to RBCs. Based on several studies, it may therefore be concluded that HA is important for viral entrance [23, 28, 30]. This compound (α-sitosterol) had an impact on NDV as the virus enters the host cell through the hemagglutinin protein, which facilitates attachment to glycoproteins and glycolipids on the cell surface [29]. Non-significant histological alterations were observed in the CAM and embryonic liver in the SPF-ECE-negative control group (α-sitosterol alone) (Figs. 4A and 5A). The epithelial and stromal layers were significantly thickened with histological changes in the NDV-inoculated ECEs. The stromal layer showed significant bleeding, clogged vasculature, and a few inflammatory cells. In addition, the epithelial layer showed epithelium that was hyperplastic, vacuolated, and had eosinophilic inclusions within the cytoplasm. Due to edema and inflammatory cell infiltration, the treated group showed significantly thickened CAM. These findings agree with those of Capua and Alexander [31].

## Conclusion

*S. misakiensis* is a promising natural antiviral agent. Its secondary metabolite (α-sitosterol) has the potential to be further developed as an antiviral medication against NDV owing to its strong antiviral activity at low doses and negligible cytotoxicity. These results demonstrate the antiviral potential of natural compounds produced from *S. misakiensis* as a source of novel antiviral agent, which contributes to the field of antiviral medication discovery. However, more research is required to examine the efficacy of α-sitosterol against different NDV serotypes, as well as possible antiviral pathways, and clinical applications.

## Methods

All methods were performed in accordance with the relevant guidelines and regulations.

### *S. misakiensis* culture and metabolites purification

To investigate the antiviral properties of *S. misakiensis* secondary metabolites *S. misakiensis* strain (GenBank accession number OP168477) was cultured in a starch nitrate broth (pH of 7.2). The broth consisted of the following components per liter: 10 g of starch, 3 g of CaCO3, 0.5 g of MgSO4, 1 g of K2HPO4, 2 g of NaNO3, and 5 g of NaCl (Oxoid, UK). Mycelium-free culture broth was obtained by combining the broth from conical flasks, each containing a *S. misakiensis* strain after incubation at 28 °C for 5 days. The filtration process involved the removal of mycelia using Büchner porcelain funnels (Stonylab, Egypt) and a coarse piece of cloth. The total volume of culture used for this procedure was 100 mL. The mycelia of *S. misakiensis* were separated by centrifugation at 13,000 rpm for 15 min. Following filtration and centrifugation, a solution containing metabolites was prepared [32].

### Characterization of *S. misakiensis* metabolites

The chemical characteristics of the metabolites were investigated using gas chromatography-mass spectrometry (GC-MS), infrared spectroscopy (IR), nuclear magnetic resonance (NMR) spectroscopy, and referencing of chemical shifts to tetramethylsilane (TMS Oxoid, UK) as an international standard.

### Thin layer chromatography

The metabolites were separated using TLC (Sigma-Aldrich, Germany) and TLC sheets. The resultant fractions were dissolved in isopropanol [33] and the bioactive metabolites were examined for their antiviral activity.

### GC mass analysis

GC-MS was used to conduct a more comprehensive examination of metabolites. The experimental setup involved the utilization of a TRACE GC Ultra Gas Chromatograph (THERMO Scientific Corp., USA). This gas chromatograph was coupled with a thermal mass spectrometer detector, specifically the ISQ Single Quadrupole Mass Spectrometer. The GC-MS system utilized a TR-5 MS column with dimensions of 30 m × 0.32 mm internal diameter and a film thickness of 0.25 μm.

The analyses were conducted using helium as the carrier gas at a flow rate of 1.0 mL/min and split ratio of 1:10. The temperature program employed for the analyses was as follows: the temperature was initially set at 60 °C for 1 min, followed by a linear increase at a rate of 4.0 °C per min until reaching 240 °C. Subsequently, the temperature was maintained at 240 °C for 1 min. The temperature of the injector and detector was maintained at 210 °C. In all experimental trials, 1 μL of the mixture was consistently injected as a diluted sample using a hexane solution with a volumetric ratio of 1:10. Mass spectra were acquired using electron ionization (EI) at an energy level of 70 eV (eV), employing a spectral range spanning from m/z 40 to 450. The chemical constituents of the metabolite were determined through deconvolution using AMDIS software (www.amdis.net). Identification was achieved by comparing retention indices to n-alkanes C8-C22, matching mass spectra to authentic standards (if available),and referencing the Wiley spectral library [34].

The NSIT library maintained by the National Institute of Standards and Technology was used for data collection and database purposes. The obtained data were confirmed using IR and NMR.

### Fourier transform infrared spectroscopy (FTIRS)

The metabolite was subjected to infrared spectroscopy (Thermo Fisher Nicolete IR IS10, USA) analysis using methanol as the solvent. The range of the spectrum is 4000–400 cm^−1^. The spectral range was assessed for its maximum and minimum resolutions as well as the number of peaks detected [33].

### Nuclear magnetic radiation spectrum (ECA-500II)

Spectra were obtained using chloroform as the solvent. NMR spectroscopy (Thermo Scientific Multiskan Sky High Microplate Spectrophotometer, Germany) is primarily employed for the purpose of obtaining accurate structural and quantitative information regarding synthesized metabolites. This is achieved using various NMR techniques, such as 1D-1H, 1D-1H decoupled 13C, 2D 1H J-resolved, 1H-1H NOESY, 1H-1H COSY, 1H-1H TOCSY, 1H-13C HSQC, and 1H-13C HMBC [35].

### Virus identification

The NDV strain (GenBank accession number MN635617) was obtained from a poultry facility located in Dakahlia Governorate, Egypt, during February 2019. This strain was isolated following consultations with the owners of the farm, who had reached the Animal Health Research Institute located in Dokki, Giza, to seek advice and discuss their prevailing circumstances. The confirmation of identification was achieved using NDV specific primers 5′-TTG ATG GCA GGC CTC TTG C-3′ and 5′-AGC GTY TCT GTC TCC T-3′ [36].

### Virus propagation and titration

The NDV strain was quantified in allantoic fluid by introducing 0.1 ml of the virus into 10-day-old SPF-ECE obtained from KomOshem SPF Farm, located in Fayoum, Egypt. Eggs were examined daily using a candling process [36]. The collection of allantoic fluid was conducted 72 h post-inoculation to determine the infectivity titers as Embryo Infective Dose 50 per mL (EID50) [37].

### Virus infectivity (EID_50_/mL)

Using SPF-ECEs, the NDV strain genotype VII (MN635617), which is predominant in the Middle East and Asia, had an infectivity titer of log10^6^ (EID_50_/mL).

## Antiviral activity of *S. misakiensis* metabolite extract

The effect of the *S. misakiensis* metabolite extract on the hemagglutination activity of NDV was assessed. A volume of 100 EID_50_/mL of the virus strain and metabolite extract were mixed and incubated at 37 °C for 30 min. The hemagglutination activity of the mixture was assessed using chicken RBCs at 0.5 and 0.75% concentrations. Phosphate-buffered saline (PBS, pH 7.2) was used as the dilution solvent [38].

### Inoculation of SPF-ECE

All methods were performed in accordance with the relevant guidelines and regulations. The study protocol was approved by the Animal-welfare ethical committee of Ain Shams University, Egypt (ASU-SCI/MICR/ 2023/9/5). After mixing 100 μL of NDV (EID_50_/mL) with 100 μL of sitosterol (0.1 mg/mL) (v/v), three twofold dilutions were prepared with dilution factors of 1/2, 1/4, and 1/8 in PBS. These dilutions were incubated at room temperature for 30 min in the presence of the NDV strain. Subsequently, 0.2 mL of the incubated dilutions were inoculated into the allantoic sacs of 10-day-old SPF-ECEs (15 eggs per group; five eggs for each dilution). Three experimental groups, α-sitosterol control group (0.02 mg /mL), NDV positive control group, and α-sitosterol- and NDV-mixture-treated group were inoculated. The ECEs were then placed in an egg incubator at 37 °C and monitored daily. After 3 days, viable eggs were kept at 4 °C. The allantoic fluids were collected to assess the NDV hemagglutination activity using RBCs at concentrations of 0.5 and 0.75% [39, 40]. The RBCs used in this study were obtained from SPF chickens. Blood was collectedfrom the jugular vein of the neck, using a sterile syringe. To prevent coagulation, 4% sodium citrate was added as the anticoagulant. The collected cells were then washed thrice with PBS at a centrifugal force of 3000 rpm. The resulting packed cells were subsequently diluted to concentrations of 0.5 and 0.75%. These diluted samples were used for rapid plate hemagglutination (HA) test. The microtiter plates used for these tests had V-bottoms and were prepared according to the guidelines outlined in the OIE Manual of Standard Diagnostic tests [39]. Following inoculation with SPF-ECE, the HA activity of NDV in allantoic fluid was assessed using 96 well microtiter plates [40].

### Histopathological examination

The CAM and liver samples obtained from the inoculated SPF-ECEs were immersed in a 10% buffered neutral formalin solution and embedded in paraffin sections. Thin sections with a thickness of 5 μm were meticulously prepared and stained with the hematoxylin and eosin (H&E) [41]. All section photos were taken using a Swift microscope (Tri-County Pkwy Schertz, TX 78154 United States) associated with Swift digital camera. The histopathological lesions were estimated by semiquantitative method [42] as follows: “0: absence of lesion, 1: 5–25%, 2: 26–50%, and 3: > 50%” (Table 1).

### Data analysis

The experiments were conducted in triplicate, and the results were reported as means ± standard deviation (SD). A Shapiro-Wilk test was performed to assess the normality assumption [43]. The data were subjected to a one-way analysis of variance.

### Supplementary Information

**Additional file 1:** **Figure S1.** The Hemagglutination activity of NDV strain (MN635617) after mixing with α-sitosterol. Three dilutions of α-sitosterol in PBS were mixed with NDV and incubated for 30 min at room temperature then tested for agglutination of 0.5% (A) and 0.75% (B) chicken RBCs (before SPF-ECE inoculation). Also, hemagglutination activity of NDV in the harvested allantoic fluid after NDV and α-sitosterol mixture inoculation in SPF-ECE inhibits agglutination of 0.5% chicken RBCs (A), and 0.75% chicken RBCs (B).

## Data Availability

All data are included in this article and in the supplementary materials.

## Abbreviations

NDV: Newcastle Disease Virus
IR: Infrared spectroscopy
GC–MS: Gas chromatography mass spectrometry
NMR: Nuclear magnetic resonance
SPF-ECEs: Specific pathogen-free embryonated chicken eggs
HA: Hemagglutination
CAM: Chorioallantoic membranes
EID50: Egg Infective Dose;
FTIRS: Fourier Transform Infrared Spectroscopy
NA: Neuraminidase
NAI: Neuraminidase inhibitors
