# Supplementary material for: Alpha-sitosterol: a new antiviral agent produced by Streptomyces misakiensis and its potential activity against Newcastle disease virus
Source: BMC Vet Res. 2024 Feb 27;20:76. doi: 10.1186/s12917-023-03875-y (PMC10898069; doi:10.1186/s12917-023-03875-y)
Supplement: Supplementary file 1 — Additional file 1: Figure S1. The Hemagglutination activity of NDV strain (MN635617) after mixing with α-sitosterol. Three dilutions of α-sitosterol in PBS were mixed with NDV and incubated for 30 min at room temperature then tested for agglutination of 0.5% (A) and 0.75% (B) chicken RBCs (before SPF-ECE inoculation). Also, hemagglutination activity of NDV in the harvested allantoic fluid after NDV and α-sitosterol mixture inoculation in SPF-ECE inhibits agglutination of 0.5% chicken RBCs (A), and 0.75% chicken RBCs (B). [file 12917_2023_3875_MOESM1_ESM.docx]

**
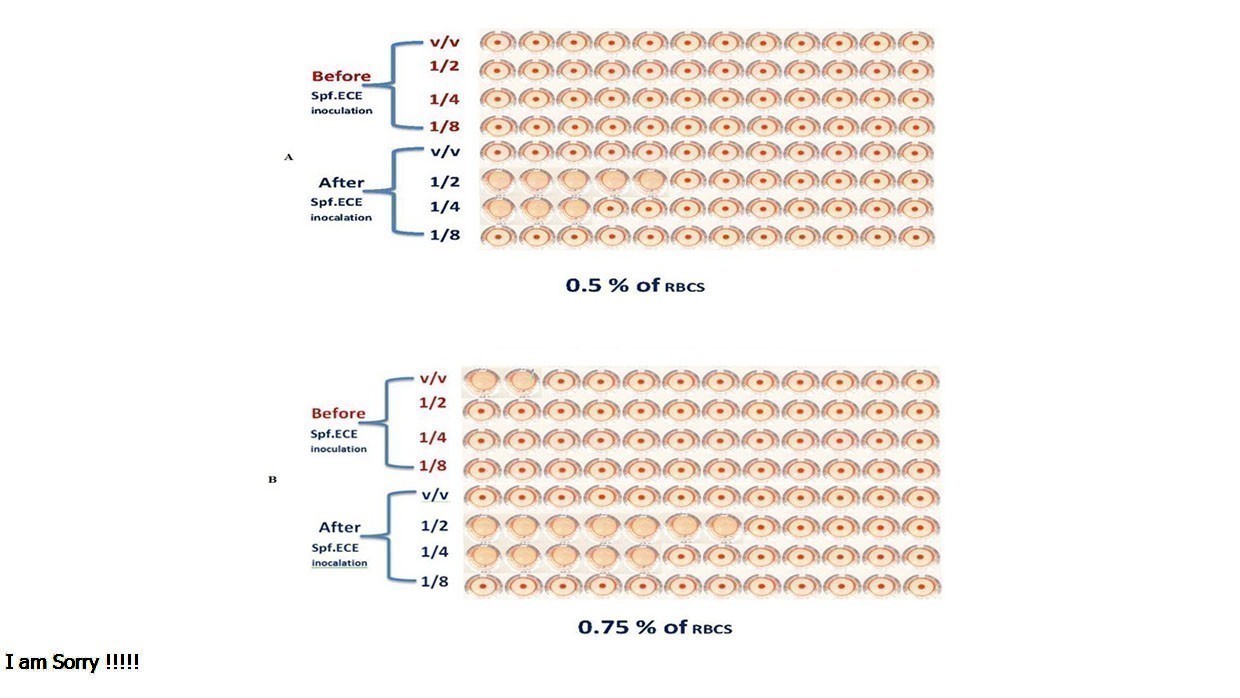
**

**Additional Figure 1**: The Hemagglutination activity of NDV strain (MN635617) after mixing with α-sitosterol. Three dilutions of α-sitosterol in PBS were mixed with NDV and incubated for 30 min at room temperature then tested for agglutination of 0.5% (A) and 0.75% (B) chicken RBCs (before SPF-ECE inoculation). Also, hemagglutination activity of NDV in the harvested allantoic fluid after NDV and α-sitosterol mixture inoculation in SPF-ECE inhibits agglutination of 0.5% chicken RBCs (A), and 0.75% chicken RBCs (B).
